# Effects of Host Plant and Insect Generation on Shaping of the Gut Microbiota in the Rice Leaffolder, *Cnaphalocrocis medinalis*

**DOI:** 10.3389/fmicb.2022.824224

**Published:** 2022-04-11

**Authors:** Yajun Yang, Xiaogai Liu, Hongxing Xu, Yinghong Liu, Zhongxian Lu

**Affiliations:** ^1^State Key Laboratory for Managing Biotic and Chemical Threats to the Quality and Safety of Agro-products, Institute of Plant Protection and Microbiology, Zhejiang Academy of Agricultural Sciences, Hangzhou, China; ^2^College of Plant Protection, Southwest University, Chongqing, China

**Keywords:** rice leaffolder, gut bacteria, host plant, Lepidoptera, rice, maize

## Abstract

Gut microbes in insects may play an important role in the digestion, immunity and protection, detoxification of toxins, development, and reproduction. The rice leaffolder *Cnaphalocrocis medinalis* (Guenée) (Lepidoptera: Crambidae) is a notorious insect pest that can damage rice, maize, and other gramineous plants. To determine the effects of host plants and generations on the gut microbiota of *C. medinalis*, we deciphered the bacterial configuration of this insect pest fed rice or maize for three generations by Illumina MiSeq technology. A total of 16 bacterial phyla, 34 classes, 50 orders, 101 families, 158 genera, and 44 species were identified in *C. medinalis* fed rice or maize for three generations. Host plants, insect generation, and their interaction did not influence the alpha diversity indices of the gut microbiota of *C. medinalis*. The dominant bacterial taxa were *Proteobacteria* and *Firmicutes* at the phylum level and *Enterococcus* and unclassified *Enterobacteriaceae* at the genus level. A number of twenty genera coexisted in the guts of *C. medinalis* fed rice or maize for three generations, and their relative abundances occupied more than 90% of the gut microbiota of *C. medinalis*. A number of two genera were stably found in the gut of rice-feeding *C. medinalis* but unstably found in the gut microbiota of maize-feeding *C. medinalis*, and seven genera were stably found in the gut of maize-feeding *C. medinalis* but unstably found in the gut of rice-feeding *C. medinalis*. In addition, many kinds of microbes were found in some but not all samples of the gut of *C. medinalis* fed on a particular host plant. PerMANOVA indicated that the gut bacteria of *C. medinalis* could be significantly affected by the host plant and host plant × generation. We identified 47 taxa as the biomarkers for the gut microbiota of *C. medinalis* fed different host plants by LEfSe. Functional prediction suggested that the most dominant role of the gut microbiota in *C. medinalis* is metabolism, followed by environmental information processing, cellular processes, and genetic information processing. Our findings will enrich the understanding of gut bacteria in *C. medinalis* and reveal the differences in gut microbiota in *C. medinalis* fed on different host plants for three generations.

## Introduction

Insects harbor numerous microorganisms in the gut (Douglas, 2015). Gut microorganisms in insects have been shown to contribute to digestion (Anand et al., 2010; Jing et al., 2020), detoxification (Ceja-Navarro et al., 2015; Beran and Gershenzon, 2016; Blanton and Peterson, 2020), development (Wang et al., 2018b; Qiao et al., 2019; Pyszko et al., 2020), physiology (Engel and Moran, 2013; Xu et al., 2019; Liberti and Engel, 2020), pathogen resistance (Dillon and Dillon, 2004; Voirol et al., 2018; Moore and Aparicio, 2022), immune response (Engel and Moran, 2013; Li et al., 2020; Li et al., 2021), and the production of essential vitamins and amino acids (Hansen and Moran, 2014; Jang and Kikuchi, 2020; Jing et al., 2020). For instance, some microorganisms with metabolic characteristics could promote insect adaptation to host plants (Voirol et al., 2018). The gut microbiota was found to function in the protection of a European *Bombus* species against the intestinal pathogen *Crithidia bombi* (Koch and Schmid-Hempel, 2011). Another example in *Helicoverpa zea* (Boddie), *Enterbacter ludwigii*, a gut-associated bacterium, could indirectly trigger the defense of tomato (*Solanum lycopersicum* L.) and maize (*Zea mays* L.) (Wang et al., 2017, 2018a). Chung et al. (2013) documented that the Colorado potato beetle *Leptinotarsa decemlineata* (Say) suppressed the defenses of tomatoes by exploiting orally secreted bacteria. The gut microbiota of the pine weevil (*Hylobius abietis*) degrades conifer diterpenes and increases insect fitness (Berasategui et al., 2017). Gut microbes may facilitate insect herbivory to chemically defend plants (Hammer and Bowers, 2015). Gut symbionts could enhance insecticide resistance in a significant pest, the oriental fruit fly *Bactrocera dorsalis* (Hendel) (Cheng et al., 2017). Insect symbionts could influence insect–plant interactions at different levels through direct interactions and also through indirect plant-mediated interactions (Frago et al., 2012). Given the importance of the associated microorganisms to host fitness and feeding ecology, an effort to manipulate these partnerships and render insect pests more vulnerable to broad-scale measures of population control by targeting the bacterial symbionts was one of the important applications in gut symbiont-driven pest control (Berasategui et al., 2016). The functions of gut microbes could provide a novel concept for the application of bacteria in pest control through the restraint of the insect immune response and the induction of plant defense (Kyritsis et al., 2017) and promote the understanding of gut symbiont-driven pest control (Frago et al., 2012; Berasategui et al., 2016).

Lepidoptera is one of the largest insect orders and has approximately 160,000 described species (Mitter et al., 2017). Some of them can damage agricultural crops and cause large economic losses (Wagner, 2013). However, the evidence of the fundamental function of bacteria in lepidopteran biology is scarce. Furthermore, a recent study from Hammer et al. (2017) reported that caterpillars lack a resident microbiome in the gut compared with other insect orders. The authors of this study argued that caterpillars with rough environments may prevent bacterial colonization. Lepidopteran reshaping the body structure during metamorphosis also enhances the difficulty of bacterial colonization (Hammer et al., 2014). Nevertheless, microbiota are abundant and diverse in many species of Lepidoptera. *Proteobacteria* and *Firmicutes* were found to be dominant in the gut of the diamondback moth *Plutella xylostella* (L.) based on the high-throughput DNA sequencing data (Xia et al., 2013). *Enterococcus* and *Lactococcus* were dominant bacteria in a field population of *Helicoverpa armigera* Hubner, followed by *Flavobacterium*, *Acinetobacter*, and *Stenotrophomonas* (Xiang et al., 2006). The composition of microbes in the insect gut could be affected by many factors. The environmental habitat, diet, developmental stage, and phylogeny of the host could determine the bacterial diversity in the insect gut (Yun et al., 2014). In the larvae of *Spodoptera littoralis* (Boisduval), bacterial communities were shown to be instar-specific (Chen et al., 2016). In addition, host plants were observed to have a considerable effect on the composition of gut bacteria in *Henosepilachna vigintioctopunctata* (F.) (Lü et al., 2019).

The rice leaffolder *Cnaphalocrocis medinalis* (Guenée) (Lepidoptera: Crambidae) is an important insect pest in Asia that can damage rice (*Oryza sativa* L.), maize, and other gramineous plants (Barrion et al., 1991; Cheng, 1996; Yang et al., 2015). The heavy occurrence of this insect could cause serious economic loss to rice production (Yang et al., 2015). In 2015, *C. medinalis* damaged rice plants with an area of 15.5 million ha and caused yield losses of 0.47 million tons in China (Yang et al., 2015; Lu, 2017). Based on the traditional isolation and culture methods, 25 species of 15 phyla of gut microbiota were obtained from *C. medinalis* larvae (Yang, 2012). By comparison, a large number of gut microbiota were obtained from *C. medinalis* larvae through Illumina MiSeq technology (Liu et al., 2016). Yang et al. (2020a) analyzed the gut microbiota composition of *C. medinalis* across the developmental stages. Information on the host-associated changes in gut bacteria will facilitate the overall understanding of insect ecology and promote the development of novel methods for pest management. This study illustrates the composition and diversity of the gut microbiota in *C. medinalis* feeding on rice or maize for three generations by Illumina MiSeq technology. The findings in this study will enrich the understanding of the gut microbiota in *C. medinalis* and provide novel insight into the relationship between *C. medinalis* and its host plants.

## Materials and Methods

### Insect Rearing and Sampling

Adults of *C. medinalis* were collected from paddy fields in Hangzhou, Zhejiang Province, East China and then cultured with 10% honey solution in the laboratory under controlled conditions of 26 ± 1°C temperature, 70 ± 10% relative humidity, and a photoperiod of 16:8 (L:D) h. The neonates of the population were divided into two groups. One was reared using rice plants, and the other was reared using maize plants. Every group was reared for three generations. Rice and maize were planted in pots (one plant per pot) in the greenhouse. The leaves of plants were collected and rinsed with sterile ddH_2_O and then air-dried before feeding them to *C. medinalis*, and sufficient leaves were provided for the insects.

Guts of *C. medinalis* were dissected from the fifth instar larvae of both groups from every generation. A total of fifteen guts were pooled into a biological sample, and three replicates were prepared for each treatment. Before dissection, the whole larva was rinsed with sterile ddH_2_O, disinfected with ethanol (75%) for 90 s, and rinsed again with sterile ddH_2_O. Following dissection, the guts were collected into a 1.5-ml sterile tube and stored at –80°C until use.

### DNA Extraction and PCR Amplification

The dissected guts were homogenized by shaking in a sterile tube containing sterile glass beads (0.5 mm diameter) and 0.5 ml of PBS buffer (pH 7.5) for 15 min using a vortex. Total DNAs were extracted from samples using the E.Z.N.A.^®^ bacteria DNA extract kit (OMEGA, United States) according to the instructions. The primers 515F 5’-GTGCCAGCMGCCGCGG-3’ and 907R 5’-CCGTCAATTCMTTTRAGTTT-3’ were used to amplify the V4-V5 regions of the bacterial 16S ribosomal RNA gene through PCR (95°C for 2 min, followed by 25 cycles at 95°C for 30 s, 55°C for 30 s, and 72°C for 30 s and a final extension at 72°C for 5 min). Amplicons were generated in a 20 μl reaction system containing 4 μl of 5 × FastPfu Buffer, 2 μl of 2.5 mM dNTPs, 0.8 μl of each primer (5 μm), 0.4 μl of FastPfu Polymerase, and 10 ng of template DNA. Blank DNA as a negative control was extracted, and products generated from no-template PCR were sequenced to assess what sequences are contaminants.

### Illumina MiSeq Sequencing

Amplicons were extracted and purified using the AxyPrep DNA Gel Extraction Kit (Axygen Biosciences, Union City, CA, United States) according to the manufacturer’s instructions and quantified using QuantiFluor™-ST (Promega, United States). Then, they were pooled in equimolar amounts and paired-end sequenced (2 × 250) on an Illumina MiSeq platform according to the standard protocols.

### Bioinformatic and Statistical Analyses

Raw FASTQ files were demultiplexed and quality-filtered using QIIME (version 1.17). According to the similarity of the sequences, effective sequences were classified into multiple operational taxonomic units (OTUs) at a similarity level of 97% using UPARSE (version 7.1),^1^ and chimeric sequences were identified and removed using UCHIME. All the sequences were annotated and blasted against the Silva (SSU115)16S rRNA database using a confidence threshold of 70% for each 16S rRNA gene sequence analyzed by RDP Classifier.^2^

Alpha diversity was estimated through five indices: OTU number, ACE, Chao1, Shannon, and Simpson’s index. The alpha diversity and relative abundance data were analyzed using one-way analysis of variance (ANOVA) with SPSS 26.0 (IBM SPSS Statistics), and multiple comparisons were analyzed using Tukey’s test. Venn diagrams and stack bars were graphed by R software. Principal coordinate analysis (PCoA) based on the matrices of pairwise weighted UniFrac distances and Bray–Curtis distances was applied among all the bacterial groups. Non-metric multidimensional scaling (NMDS) plots were constructed using Bray–Curtis. Analysis of similarity (ANOSIM) was used to test the difference in the composition of microbiota among different group samples. Permutational multivariate analysis of variance (PerMANOVA) was generated using 999 permutations, and the individual repeats were included in the model as a random effect. PCoA, NMDS, ANOSIM, and PerMANOVA were analyzed and graphed using R software. Linear discriminant analysis (LDA) was used to screen the biomarkers for significant differences between different groups with LDA scores greater than two. A cladogram was drawn to show the distribution of these biomarkers at different taxonomic levels by Galaxy (accessed on 1 January 2022).^3^ Microbiota functions were predicted by annotating pathways of OTUs against the Ref99NR database using R software with the Tax4Fun2 package.

## Results

### Reads Analyzed and Taxa Generated

We sequenced the gut microbes of *C. medinalis* fed on different host plants for three generations and obtained 1,473,836 trimmed paired reads in total (Supplementary Table 1). Blank DNA and no-template PCR sequencing were used for decontamination, and sequences of cyanobacteria or chloroplasts were found to be contaminants. After decontamination, 446 OTUs were obtained. The OTU numbers of *C. medinalis* from different samples varied from 49 to 194 (Table 1). The Ace index varied from 62.93 to 252.14, the Chao1 index varied from 57.27 to 256.25, the Shannon index varied from 0.47 to 1.36, and the Simpson index varied from 0.46 to 0.87 (Table 1). ANOVA indicated that alpha diversity indices were not significantly affected by the host plant, generation, or their interaction (Supplementary Table 2). A total of 16 bacterial phyla, 34 classes, 50 orders, 101 families, 158 genera, and 44 species were identified in *C. medinalis* fed rice or maize for three generations (Table 2).

**TABLE 1:** Alpha diversity indices of gut bacterial communities in rice- or maize-feeding *Cnaphalocrocis medinalis* for three generations.

Sample*^a^*	Alpha diversity indices				
	OTU number	ACE	Chao1	Shannon	Simpson
R1-1	201	252.14	256.25	1.33	0.60
R1-2	59	85.09	75.15	0.88	0.60
R1-3	49	62.93	57.27	0.58	0.77
R2-1	162	210.16	225.14	0.52	0.84
R2-2	157	194.09	200	0.47	0.87
R2-3	183	221.45	228.12	0.90	0.62
R3-1	85	107.55	110.07	0.95	0.59
R3-2	61	117.04	119	0.94	0.56
R3-3	145	178.45	184.26	1.36	0.46
M1-1	194	242.48	231.66	1.20	0.52
M1-2	69	95.25	81.67	0.81	0.65
M1-3	135	184.21	197.67	0.52	0.85
M2-1	171	207.09	212.25	1.17	0.52
M2-2	165	189	180.53	1.26	0.46
M2-3	159	194.74	222.07	1.15	0.58
M3-1	96	120.32	113.25	0.95	0.52
M3-2	110	144.6	153.5	0.94	0.58
M3-3	98	108.44	111.91	0.81	0.59

*^a^R1–R3: the first to third generation of C. medinalis fed on rice; M1–M3: the first to third generation of C. medinalis fed on maize.*

**TABLE 2:** Number of identified gut bacterial taxonomic categories in rice- and maize-feeding *Cnaphalocrocis medinalis* for three generations.

Treatments*^a^*	Phylum	Class	Order	Family	Genus	Species
R1	12	28	39	69	99	30
R2	13	30	42	86	118	36
R3	11	23	33	68	93	30
M1	13	29	40	74	106	32
M2	12	26	42	85	115	37
M3	5	9	21	51	71	31
Total	16	34	50	101	158	44

*^a^R1–R3, the first to third generation of C. medinalis fed rice; M1–M3, the first to third generation of C. medinalis fed maize.*

### Gut Microbiota of *Cnaphalocrocis medinalis* Fed Rice for Three Generations

**FIGURE 1:**
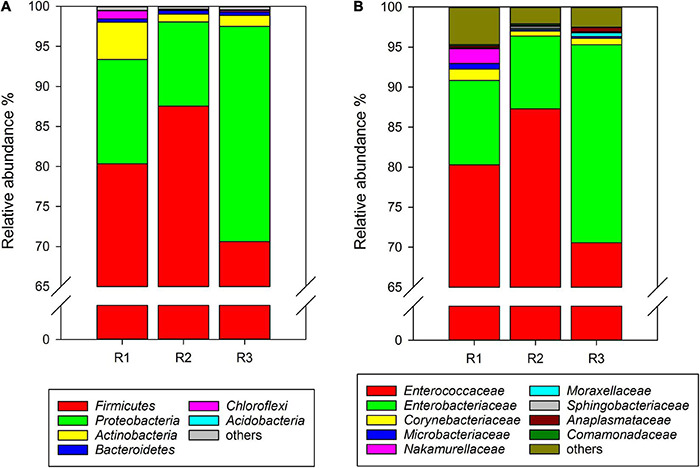
Relative abundance of the gut microbiota from rice-feeding *C. medinalis* for three generations at the phylum **(A)** and family **(B)** levels. R1–R3: the first to third-generation of *C. medinalis* fed on rice.

At the phylum level, *Firmicutes*, *Proteobacteri*a, *Actinobacteria*, *Bacteroidetes*, and unclassified *Bacteria* were found in the gut microbiota of *C. medinalis* fed on rice plants through all samples of three generations. Among them, *Firmicutes* was the absolute dominant phylum with the highest relative abundance in rice-feeding *C. medinalis* for three generations (70.62–87.53%) (Figure 1A). The relative abundance of *Proteobacteria* was 10.51–26.88%, followed by *Actinobacteria* (1.00–4.66%), *Bacteroidetes* (0.41–0.43%), and unclassified *Bacteria* (0.02–0.14%) (Figure 1A). At the family level, 18 families were found in the gut microbiota of *C. medinalis* fed rice through all samples of three generations. *Enterococcaceae* and *Enterobacteriaceae* were the two major families in the rice-feeding *C. medinalis* for three generations, with relative abundance ranges of 70.55–87.27% and 9.12–24.75%, respectively (Figure 1B). The relative abundance of *Anaplasmataceae* in the gut of the third generation of *C. medinalis* fed rice was higher than that of the second generation of *C. medinalis*, and the relative abundance of *Nocardiaceae* in the gut of the first generation of *C. medinalis* fed rice was higher than that of the other two generations of *C. medinalis* (Figure 1B). At the genus level, 21 genera were found in the gut microbiota of *C. medinalis* fed rice through all samples of three generations. *Enterococcus*, unclassified *Enterobacteriaceae*, *Pectobacterium*, *Corynebacterium*, *Leucobacter*, and *Anaplasma* occupied the top 10 in the gut microbiota of *C. medinalis* fed rice for three generations (Supplementary Table 3). Common genera found in all three generations occupied 93.95, 98.51, and 97.78% of the first generation to the third generation, respectively. *Enterococcus* and unclassified *Enterobacteriaceae* are the majority. In addition to the microbes found in all samples of rice-feeding *C. medinalis* for three generations, many kinds of microbes were found in some but not all samples of rice-feeding *C. medinalis* gut.

### Gut Microbiota of *Cnaphalocrocis medinalis* Fed Maize for Three Generations

**FIGURE 2:**
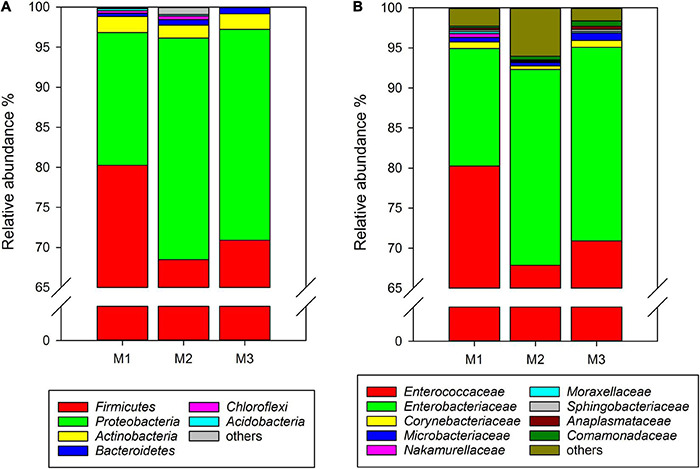
Relative abundance of the gut microbiota from maize-feeding *C. medinalis* for three generations at the phylum **(A)** and family **(B)** levels. M1–M3: the first to third generation of *C. medinalis* fed on maize.

Similar to rice-feeding *C. medinalis*, the same six phyla were found in the gut microbiota of maize-feeding *C. medinalis* through all samples of three generations. The phylum with the highest relative abundance was *Firmicutes* (68.49–80.25%), followed by *Proteobacteria* (16.58–27.65%), *Actinobacteria* (1.62–2.01%), *Bacteroidetes* (0.41–0.83%), and unclassified *Bacteria* (0.004–0.29%) (Figure 2A). At the family level, 20 families found gut microbiota of *C. medinalis* fed maize through all samples of three generations. *Enterococcaceae* and *Enterobacteriaceae* were also the dominant families, with relative abundance ranges of 67.88–80.23% and 14.69–24.42%, respectively (Figure 2B). The relative abundance of *Comamonadaceae* in the gut of the third generation of *C. medinalis* fed maize was higher than that of the first generation of *C. medinalis*, the relative abundance of *Micrococcaceae* in the second generation of *C. medinalis* was higher than that of the first generation of *C. medinalis*, and the relative abundance of *Rhodocyclaceae* in the gut of the second generation of *C. medinalis* was higher than that of the third generation of *C. medinalis* (Supplementary Table 3). At the genus level, 26 genera were found in the gut microbiota of *C. medinalis* fed maize through all samples of three generations. *Enterococcus*, unclassified *Enterobacteriaceae*, *Corynebacterium*, unclassified *Comamonadaceae*, *Leucobacter*, *Microbacterium*, *Anaplasma*, and *Sphingobacterium* occupied the top 10 in the gut microbiota of *C. medinalis* fed maize for three generations (Supplementary Table 3). Common genera found in all three generations occupied 97.75, 96.34, and 99.29% of the first generation to the third generation, respectively. *Enterococcus* and unclassified *Enterobacteriaceae* are the majority. In addition to the microbes found in all samples of maize-feeding *C. medinalis* for three generations, many kinds of microbes were found in some but not all samples of maize-feeding *C. medinalis* gut.

### Influence of Host Plant and Insect Generation on the Gut Bacterial Communities of *Cnaphalocrocis medinalis*

**FIGURE 3:**
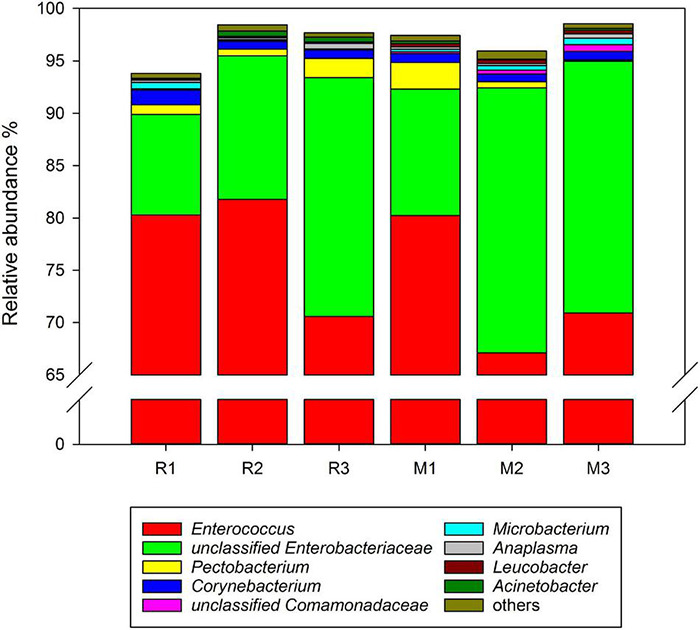
Relative abundance of the genera in the gut microbiotas found in all samples of *C. medinalis*. R1–R3: the first to third generation of *C. medinalis* fed rice; M1–M3: the first to third generation of *C. medinalis* fed maize.

Comparing the gut microbiota between *C. medinalis* fed rice and maize, five phyla and 16 families were found in all samples of the three generations. At the genus level, 19 genera were found in the gut microbiota of *C. medinalis* fed on rice or maize plants for three generations (Supplementary Table 3). The relative abundance of these genera occupied more than 90% of the gut microbiota of *C. medinalis* fed rice or maize plants, and the two major genera were *Enterococcus* and unclassified *Enterobacteriaceae* (Figure 3). Seven genera, *Bacillus*, *Empedobacter*, *Flavobacterium*, *Rhizobium*, *Rhodococcus*, *Sphingobacterium*, and unclassified *Beutenbergiaceae*, were stably found in all samples of maize-feeding *C. medinalis* for three generations, whereas *Tsukamurella* and *Ochrobactrum* were stably found in all samples of rice-feeding *C. medinalis* for three generations (Supplementary Table 4).

**FIGURE 4:**
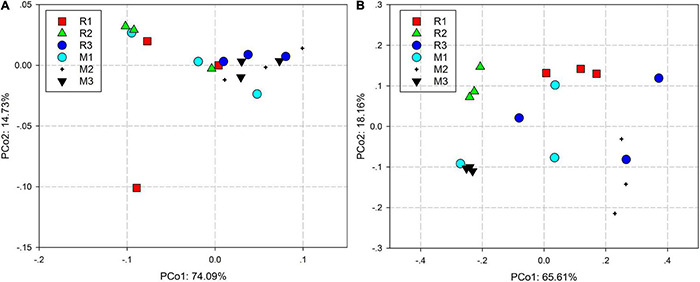
PCoA bacterial communities of *C. medinalis* fed on different host plants over generations based on weighted UniFrac **(A)** and Bray–Curtis **(B)** distances. M1–M3: the first to third generation of *C. medinalis* fed on maize; R1–R3: the first to third generation of *C. medinalis* fed on rice.

Principal coordinate analysis based on the Bray–Curtis distance and weighted UniFrac distance was used to compare the community similarities between samples. The PCoA scatter plot showed that the abscissa and ordinate represent the two characteristic values that contribute to the largest differences between the samples, and their influence degrees were 74.09 and 14.73% based on weighted UniFrac distance (Figure 4A) and 65.61 and 18.16% based on the Bray–Curtis (Figure 4B), respectively. PerMANOVA showed that there were significant differences in the gut microbiota of rice- and maize-feeding *C. medinalis* (Table 3; PerMANOVA: *R*^2^ = 0.35, *p* = 0.001). Host plant × generation significantly affected the gut microbiota of *C. medinalis* (Table 3; PerMANOVA: *R*^2^ = 0.28, *p* = 0.004). No significant differences were observed between the samples from different generations of *C. medinalis* (Table 3; PerMANOVA: *R*^2^ = 0.02, *p* = 0.751).

**TABLE 3:** PERMANOVA of the bacterial communities of *C. medinalis* fed rice or maize for three generations.

Source	*df*	*SS*	*MS*	Pseudo-F	*R* ^2^	*p*-value
Host plant	1.17	1.5924	1.5924	8.66	0.35	0.001
Generation	1.17	0.1086	0.1086	0.39	0.02	0.751
Host plant × Generation	1.17	1.2699	1.2699	6.22	0.28	0.004

*PERMANOVA was generated using 999 permutations, and the individual repeat was included in the model as a random effect.*

**FIGURE 5:**
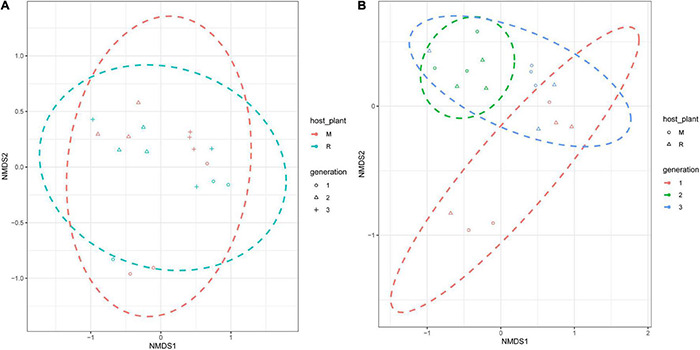
NMDS analysis of the bacterial communities of *C. medinalis* fed maize and rice for three generations. NMDS plots were constructed using Bray–Curtis with the main groups of host plants **(A)** and generations **(B)**. Stress values (stress = 0.079) indicate a good fit in two dimensions. M: samples from *C. medinalis* fed maize; R: samples from *C. medinalis* fed rice.

Non-metric multidimensional scaling analysis revealed significant differences between the gut microbiota of rice- and maize-feeding *C. medinalis* (Figure 5). ANOSIM showed that there were significant differences in the gut microbiota of rice- and maize-feeding *C. medinalis* (*R* = 0.5538, *p* = 0.001) (Supplementary Table 5). There were no significant differences in the gut microbiota of rice- and maize-feeding *C. medinalis* in the same generations (Supplementary Table 5).

**FIGURE 6:**
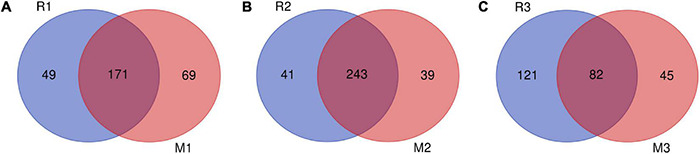
Venn diagram of bacterial community OTUs of *C. medinalis* fed different host plants for three generations. M1–M3: the first to third generation of *C. medinalis* fed on maize; R1–R3: the first to third generation of *C. medinalis* fed on rice. **(A)** M1 vs. R1; **(B)** M2 vs. R2; **(C)** M3 vs. R3.

Venn diagrams showed overlapping OTUs of *C. medinalis* fed on rice or maize from the first generation to the third generation (Figure 6). The results indicated that 171 OTUs, which comprised 77.73 and 71.25% of the total OTUs of the first generation of *C. medinalis* fed rice or maize, were shared by *C. medinalis* fed rice or maize (Figure 6A). The second generation of *C. medinalis* fed on rice or maize shared 243 OTUs, which accounted for 85.56 and 86.17% of the total OTUs of the second generation of *C. medinalis* fed on rice or maize, respectively (Figure 6B). The third generation of *C. medinalis* fed on rice or maize shared 82 OTUs, which accounted for 40.39 and 64.57% of the total OTUs of the third generation of *C. medinalis* fed on rice or maize, respectively (Figure 6C).

**FIGURE 7:**
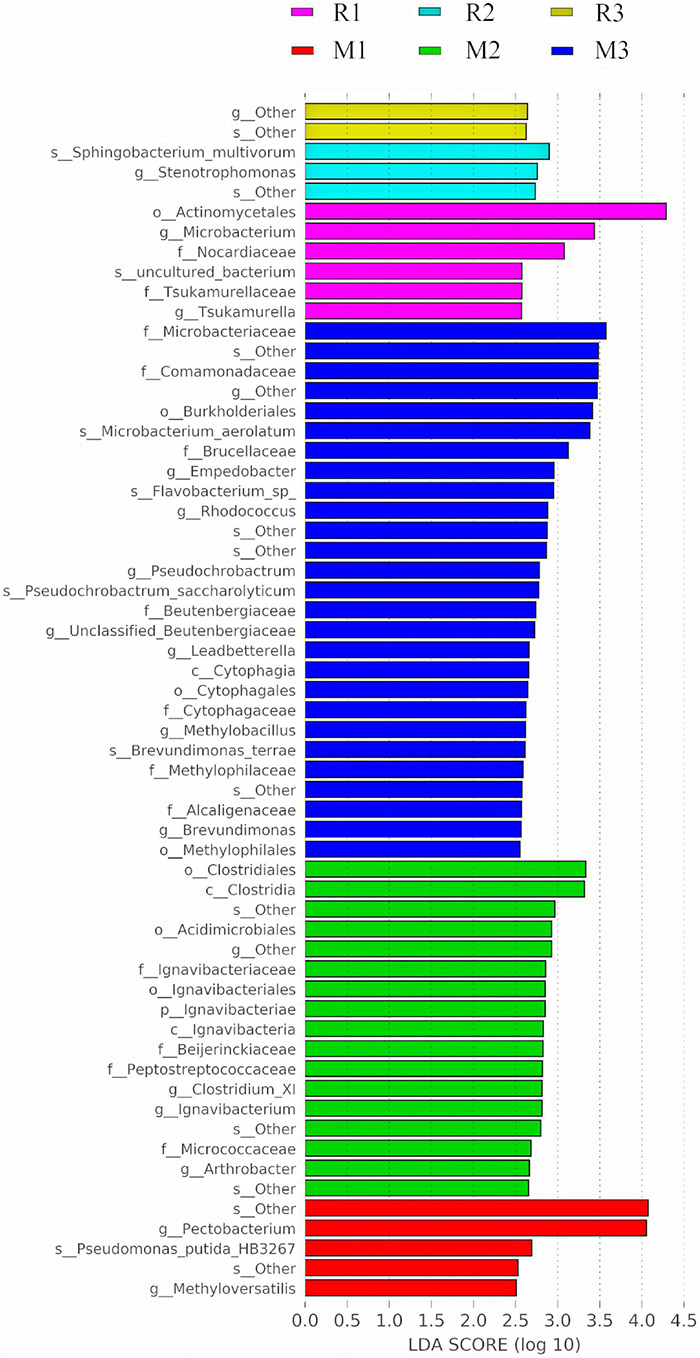
Bacterial taxa with LDA scores greater than two in the gut microbiota of *C. medinalis* fed different host plants for three generations.

**FIGURE 8:**
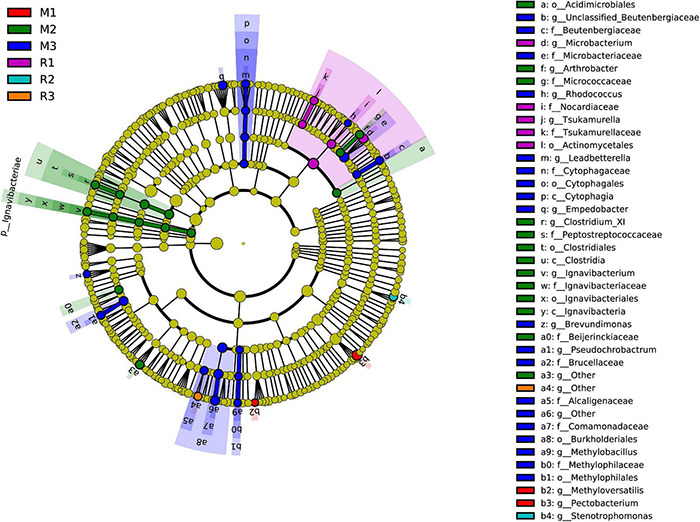
Cladogram of bacterial biomarkers, from the phylum (innermost ring) to genus (outermost ring) level, with an LDA score > 2. Differential bacterial taxa are marked by lowercase letters. Each small circle at different taxonomic levels represents a taxon at that level, and the diameter of the circle is proportional to the relative abundance. The coloring principle is to color the species with no significant difference as yellow and the other different species as the group with the highest abundance of the species. Different colors represent different groups, and nodes with different colors represent the communities that play an important role in the group represented by the color.

To find the biomarkers with significant differences between different groups, LDA effect size (LEfSe) was used to screen out different taxa at various levels (kingdom, phylum, class, order, family, genus, and species) between different groups based on a standard LDA value greater than two (Figure 7). Meanwhile, the cladogram from phylum to genus was graphed to fully understand the distribution of these different taxa at various taxonomic levels (Figure 8). In the third generation of *C. medinalis* fed maize (M3), the gut microbiota had the most of the different taxa (LDA > 2). There were 60 taxa mainly belonging to *Firmicutes*, *Bacteroidota*, *Acidobacteriota*, *Proteobacteria*, *Actinobacteria*, and *Ignavibacteriae*. A total of six taxa belonging to *Actinobacteria* were in the gut microbiota of the first generation of *C. medinalis* fed rice (R1). A total of two taxa belonging to *Proteobacteria* and one taxon belonging to *Bacteroidetes* were in the gut microbiota of the second generation of *C. medinalis* fed rice (R2). A total of two taxa belonging to *Proteobacteria* were in the gut microbiota of the third generation of *C. medinalis* fed rice (R3). A total of five taxa belonging to *Proteobacteria* were in the gut microbiota of the first generation of *C. medinalis* fed maize (M1). A total of three taxa belonging to *Proteobacteria*, five taxa exclusive to *Ignavibacteriae*, five taxa belonging to *Firmicutes*, and four taxa belonging to *Actinobacteria* were in the gut microbiota of the second generation of *C. medinalis* fed maize (M2). A total of fourteen taxa belonging to *Proteobacteria*, seven taxa belonging to *Actinobacteria*, and six taxa belonging to *Bacteroidetes* were in the gut microbiota of the third generation of *C. medinalis* fed on maize (M3). LEfSe was also used to find the biomarkers with significant differences between samples fed different host plants (Supplementary Figure 1). A total of forty-seven taxa were identified as the biomarkers in the gut microbiota of *C. medinalis* fed on different host plants (Supplementary Figure 2). A total of six taxa belonging to *Actinobacteria* and one taxon belonging to *Proteobacteria* were in the gut microbiota of *C. medinalis* fed rice. Nineteen taxa belonging to *Proteobacteria*, 10 taxa belonging to *Bacteroidetes*, and 11 taxa belonging to *Actinobacteria* were in the gut microbiota of *C. medinalis* fed maize.

### Functional Prediction of the Gut Microbitota of *Cnaphalocrocis medinalis*

**FIGURE 9:**
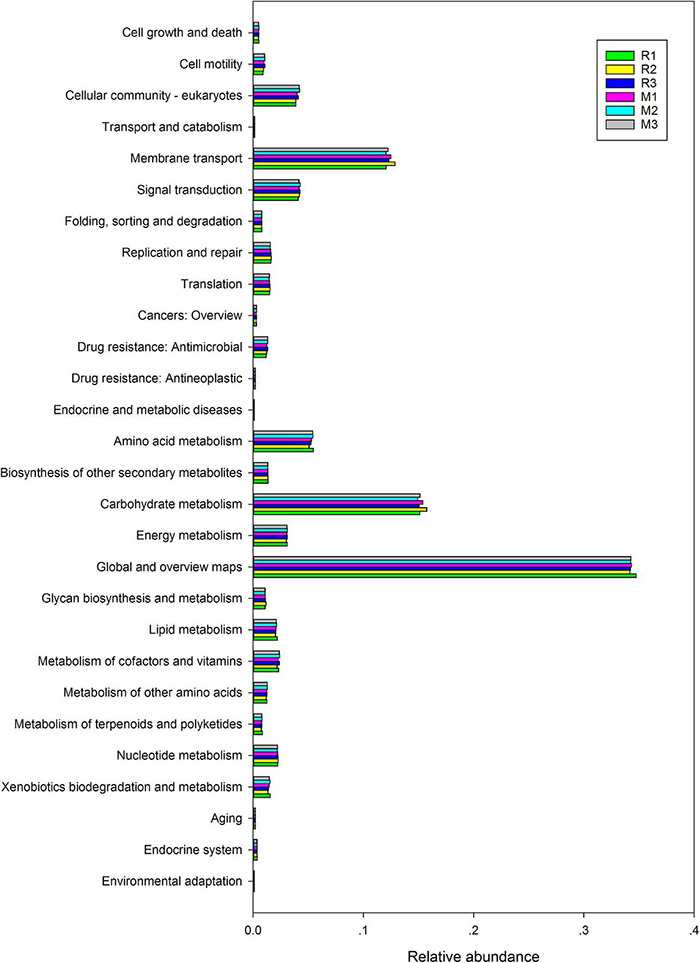
Comparison of predicted GO functions of the gut bacteria of *C. medinalis* fed different host plants for three generations.

To better understand the important role of the gut microbiota of *C. mednialis*, we used R software with Tax4Fun2 to predict the function in samples based on 16S rDNA sequencing data and compared them with the Ref99NR database (Figure 9). The results showed that the most functional prediction categories were related to metabolism (70.12–71.18%) followed by environmental information processing (16.17–17.08%), cellular processes (5.39–5.94%), and genetic information processing (3.87–4.06%). In the metabolism category, global and overview maps had the highest abundance (34.18–34.73%) followed by carbohydrate metabolism (14.93–15.75%), amino acid metabolism (5.08–5.45%), energy metabolism (3.00–3.10%), metabolism of cofactors and vitamins (2.16–2.40%), nucleotide metabolism (2.18–2.26%), lipid metabolism (2.02–2.17%), xenobiotics biodegradation and metabolism (1.34–1.55%), biosynthesis of other secondary metabolites (1.31–1.35%), metabolism of other amino acids (1.12–1.28%), glycan biosynthesis and metabolism (1.08–1.14%), and metabolism of terpenoids and polyketides (0.74–0.85%). In the environmental information processing category, membrane transport had the highest abundance (12.05–12.87%) followed by signal transduction (4.08–4.26%). In the cellular processes category, the cellular community had the highest abundance (3.85–4.21%) followed by cell motility (0.88–1.06%), cell growth and death (0.49–0.52%), and transport and catabolism (0.14–0.15%). In the genetic information processing category, replication and repair had the highest abundance (1.55–1.64%), followed by translation (1.45–1.53%) and folding, sorting, and degradation (0.76–0.79%).

## Discussion

This study profiled the gut bacterial community in *C. medinalis* fed on different host plants for three generations. Considering the limited gut bacterial information isolated and cultured by traditional methods (Yang, 2012), we obtained the bacterial information of *C. medinalis* by MiSeq sequencing. Recently, we reported that the composition of the gut bacterial community changes across the life cycle of *C. medinalis*, and the phyla *Proteobacteria* and *Firmicutes* were the dominant bacterial taxa (Yang et al., 2020a). In the guts of both *C. medinalis* fed rice and maize, the phyla *Proteobacteria* and *Firmicutes* were also the dominant bacterial taxa. In this study, host plants, generation, and their interaction did not significantly affect the alpha diversity indices of the gut microbiota in *C. medinalis*. Ace and choa1 values indicated that community richness did not differ among the different groups. Shannon and Simpson values indicated that community diversity did not differ among the different groups. The experimental results provide a more comprehensive understanding of the relationship between *C. medinalis* and its microbiota. Our results revealed the influence of host plants and insect generation on the gut bacterial community in *C. medinalis* and provide a foundation for investigating gut microbe *C. medinalis*–host plant interactions.

Diet is one of the important factors for insect development (Karley et al., 2002; Qubaiová et al., 2021), and it also plays an important role in shaping insect phenotypes and gut microbial communities (Colman et al., 2012; Xu et al., 2019; Luo et al., 2021; Mason et al., 2021). Host diet could influence the diversity, structure, or composition of the gut in many insects (Strano et al., 2018; Lü et al., 2019; Leite-Mondin et al., 2021; Yuan et al., 2021). Leite-Mondin et al. (2021) discovered that the gut microbiota composition of *Trichoplusia ni* (Hubner) altered by diet may influence its polyphagous behavior. An imbalanced diet-altered variation in gut microbiota is detrimental to mirid bugs, *Adelphocoris suturalis* Jakovlev (Luo et al., 2021). In this study, at the family and genus levels, the composition of the gut microbiota of *C. medinalis* differed between the host plants. Among the genera found in the gut of *C. medinalis* fed different host plants, only 21 genera were found in all samples of three generations of rice-feeding *C. medinalis*, and only 26 genera were found in all samples of three generations of maize-feeding *C. medinalis*. These results indicated that most kinds of microbes are not stably colonized in the gut of *C. medinalis* fed a particular host plant. Hammer et al. (2017) reported that caterpillars lack a resident gut microbiome. Jones et al. (2019) found high variability in gut bacterial composition and abundance between the individuals of the same insect species even fed on the same food source. The reports from other lepidopteran species showed that gut microbial assemblages differed between individuals (Priya et al., 2012; Staudacher et al., 2016). In this study, only 19 genera coexisted in *C. medinalis* fed rice or maize, whereas their relative abundances occupied more than 90% of the gut microbiota of *C. medinalis* fed rice or maize. In addition, we found that two genera (*Tsukamurella* and *Ochrobactrum*) were stable in the gut of rice-feeding *C. medinalis*, but unstable in the gut microbiota of maize-feeding *C. medinalis*, and seven genera (*Bacillus*, *Empedobacter*, *Flavobacterium*, *Rhizobium*, *Rhodococcus*, *Sphingobacterium*, and unclassified *Beutenbergiaceae*) were stable in the gut of maize-feeding *C. medinalis*, but unstable in the gut of rice-feeding *C. medinalis*. For example, some genera that were stable in the gut of maize-feeding *C. medinalis* were found in some but not all samples of rice-feeding *C. medinalis*. The gut bacteria that were stable in the gut of *C. medinalis* for three generations may have an important role in shaping the microbiota community in *C. medinalis*. Through LEfSe, 47 taxa were found to be the biomarkers for the gut microbiota of *C. medinalis* fed different host plants. Stable host-related bacteria may function to help *C. medinalis* to adapt to host plants. In addition to diet, there are many factors that influence the gut microbiota in insects. Life stage and environment could shape the insect gut microbial community combined with diets as drivers (Colman et al., 2012). Host plant and population sources could drive the diversity of the microbial community in two polyphagous insects (Jones et al., 2019). Different host genotypes and microbial sources could influence the gut bacterial communities in lepidopterans (Mason et al., 2021). In this study, host plant × insect generation may be a factor influencing the gut microbiota in *C. medinalis*. In the colonization of gut microbes, the interaction of the host plant and generation may play an important role. A recent study indicated that diet is not the primary driver of gut bacterial community structure in wood- and litter-feeding cockroaches (Lampert et al., 2019). The phyllosphere microbiome in host plants contributes more than leaf phytochemicals to the variation in the gut microbiome structure in *Agrilus planipennis* (Mogouong et al., 2021). In lepidopterans, metamorphosis, which entails major morphological changes with dietary transformation, could also have a strong impact on the gut microbiota composition (Voirol et al., 2018). However, certain taxa can persist throughout all the stages of the insect (Hammer et al., 2014; Yang et al., 2020a). In insects, the gut microbiota can promote gut homeostasis (Buchon et al., 2013), and core microbes in the gut microbiota may reach homeostasis by interacting with the factors in the environment. Gut microbes coexisting in all samples of rice- and maize-feeding *C. medinalis* may compose the core microbes in *C. medinalis*.

The gut microbiota could play a crucial role in the whole life of insects. The lepidopteran gut microbiota could function in digestion and nutrient acquisition, protection against entomopathogens, and counteraction to anti-herbivore plant defenses (Voirol et al., 2018). Jing et al. (2020) found that the most dominant role of gut bacteria is essential nutrient provisioning, followed by digestion and detoxification. In this study, functional prediction indicated that the most dominant role of the gut microbiota in *C. medinalis* is metabolism, followed by environmental information processing, cellular processes, and genetic information processing. Distinct antimicrobials could alter gut microbial communities as a result of different mortalities of *P. xylostella* (Lin et al., 2015). The gut microbiota involved in *P. xylostella* susceptibility to Bt Cry1Ac protoxin is associated with the host immune response (Li et al., 2021). In the guts of both *C. medinalis* fed rice and maize, the *Proteobacteria* and *Firmicutes* phyla were the dominant bacterial taxa. *Proteobacteria* and *Firmicutes* have also been reported as dominant taxa in many insects’ gut microbiota, especially in Lepidoptera (Chen et al., 2020; Liu et al., 2020). They may function in carbohydrate metabolism, amino acid metabolism, and membrane transport pathways of the host (Liu et al., 2020; Wang et al., 2020a; Chen et al., 2021). In particular, stably colonized gut bacteria may be crucial for insects to adapt to host plants (Yang et al., 2020b). Global and overview maps, carbohydrate metabolism, membrane transport, amino acid metabolism, signal transduction, and cellular community were the top six pathways in the functions of the gut microbiota in *C. medinalis*. *Enterococcus* is an important flora that exists in both rice- and maize-feeding *C. medinalis* for three generations, followed by the unclassified *Enterobacteriaceae*, *Pectobacterium*, and *Corynebacterium*. *Enterococcus* has also been reported to be stably maintained in many insects, and it can protect insects against pathogens, fix toxic molecules from plants, increase host fitness, and tolerate toxic diets (Shao et al., 2011; Johnston and Rolff, 2015; Vilanova et al., 2016; Shao et al., 2017). *Enterobacteriaceae* is one of the important dominant taxa in the gut microbiota of many insects (Wang et al., 2014; Yun et al., 2018; Raza et al., 2020). *Enterobacteriaceae* are involved in insect metabolism (Pers and Hansen, 2021; Zhou et al., 2021), insect resistance or susceptibility to parasites, and pathogens and insecticides (Oliver et al., 2003; Álvarez-Lagazzi et al., 2021; Polenogova et al., 2021) and play an important role in the host adaptability and reproduction of insects (Shi et al., 2012; Wang et al., 2020b). *Pectobacterium*, a clade of *Enterobacteriaceae*, is known as a function of nitrogen fixation (Behar et al., 2005, 2008). In addition to fixing nitrogen, the gut microbiota may help recycle nitrogenous waste products into usable compounds, such as uric acid and ammonia (Behar et al., 2005, 2008). *Corynebacterium*-related bacteria grow on a variety of sugars, organic acids, and alcohols as the single or combined carbon and energy sources as a workhorse for the large-scale production of amino acids (Eikmanns and Blombach, 2014). The detailed actual functions of these microbes in the gut of *C. medinalis* need to be proven and verified in further investigations.

## Conclusion

In conclusion, our results indicated that the alpha diversity indices of gut microbes in *C. medinalis* could not be affected by the host plant, generation, or host plant × generation. PerMANOVA indicated that the gut bacteria of *C. medinalis* could be significantly affected by the host plant and host plant × generation. Coexisting bacteria that were found in both rice- and maize-feeding *C. medinalis* for three generations may play an important role in the development of insects, while stably colonized bacteria in *C. medinalis* fed a particular plant may function in host adaptation. The most dominant role of the gut microbiota in *C. medinalis* is metabolism, followed by environmental information processing, cellular processes, and genetic information processing. Furthermore, further experiments should be performed to reveal the function of these microbes, which may promote the identification of new targets for the management of *C. medinalis*. Our results provide a theoretical basis for the study of gut microbes in *C. medinalis*.

## Data Availability Statement

The datasets presented in this study can be found in online repositories. The names of the repository/repositories and accession number(s) can be found below: NCBI—PRJNA785679, SRR17106748–SRR17106753

## Author Contributions

YY, YL, and ZL contributed to conceptualization of the study. YY and ZL contributed to funding acquisition. XL investigated the study. HX contributed to methodology. YL and ZL contributed to supervision. YY and XL visualized the study. YY wrote the original draft and contributed to writing, reviewing, and editing the manuscript. All authors have read and agreed to the published version of the manuscript.

## Conflict of Interest

The authors declare that the research was conducted in the absence of any commercial or financial relationships that could be construed as a potential conflict of interest.

## Publisher’s Note

All claims expressed in this article are solely those of the authors and do not necessarily represent those of their affiliated organizations, or those of the publisher, the editors and the reviewers. Any product that may be evaluated in this article, or claim that may be made by its manufacturer, is not guaranteed or endorsed by the publisher.

## Footnotes

1
http://drive5.com/uparse/

2
http://rdp.cme.msu.edu/

3
http://huttenhower.sph.harvard.edu/galaxy/

## Supplementary Material

Supplementary Figure 1 Bacterial taxa with linear discriminant analysis (LDA) score >2 in the gut microbiota of *C. medinalis* fed on different host plants.

Supplementary Figure 2 Cladogram of bacterial biomarkers, from the phylum (innermost ring) to genus (outermost ring) level, with an LDA score >2. Differential bacterial taxa are marked by lowercase letters. Each small circle at different taxonomic levels represents a taxon at that level, and the diameter of the circle is proportional to the relative abundance. The coloring principle is to color the species with no significant difference as yellow and the other different species as the group with the highest abundance of the species. Different colors represent different groups, and nodes with different colors represent the communities that play an important role in the group represented by the color.

Supplementary Table 1 Sequencing statistics of gut microbiota from rice- or maize-feeding *Cnaphalocrocis medinalis* for three generations.

Supplementary Table 2 ANOVA of Alpha diversity indices of gut bacterial communities in *Cnaphalocrocis medinalis* fed rice or maize for three generations.

Supplementary Table 3 Relative abundance (%) of genus in gut microbiota of rice- or maize-feeding *Cnaphalocrocis medinalis* for three generations.

Supplementary Table 4 List of genera of the gut microbiota only stable in all samples of rice- or maize-feeding *Cnaphalocrocis medinalis.*

Supplementary Table 5 Analysis of similarity (ANOSIM) between gut microbial communities from within sample groups of *Cnaphalocrocis medinalis*.
