# Supplementary material for: Effects of Host Plant and Insect Generation on Shaping of the Gut Microbiota in the Rice Leaffolder, Cnaphalocrocis medinalis
Source: Front Microbiol. 2022 Apr 11;13:824224. doi: 10.3389/fmicb.2022.824224 (PMC9037797; doi:10.3389/fmicb.2022.824224)
Supplement: Supplementary Figure 1 — Bacterial taxa with linear discriminant analysis (LDA) score >2 in the gut microbiota of C. medinalis fed on different host plants. [file Data_Sheet_1.ZIP › Supplementary Figure 2.pdf]

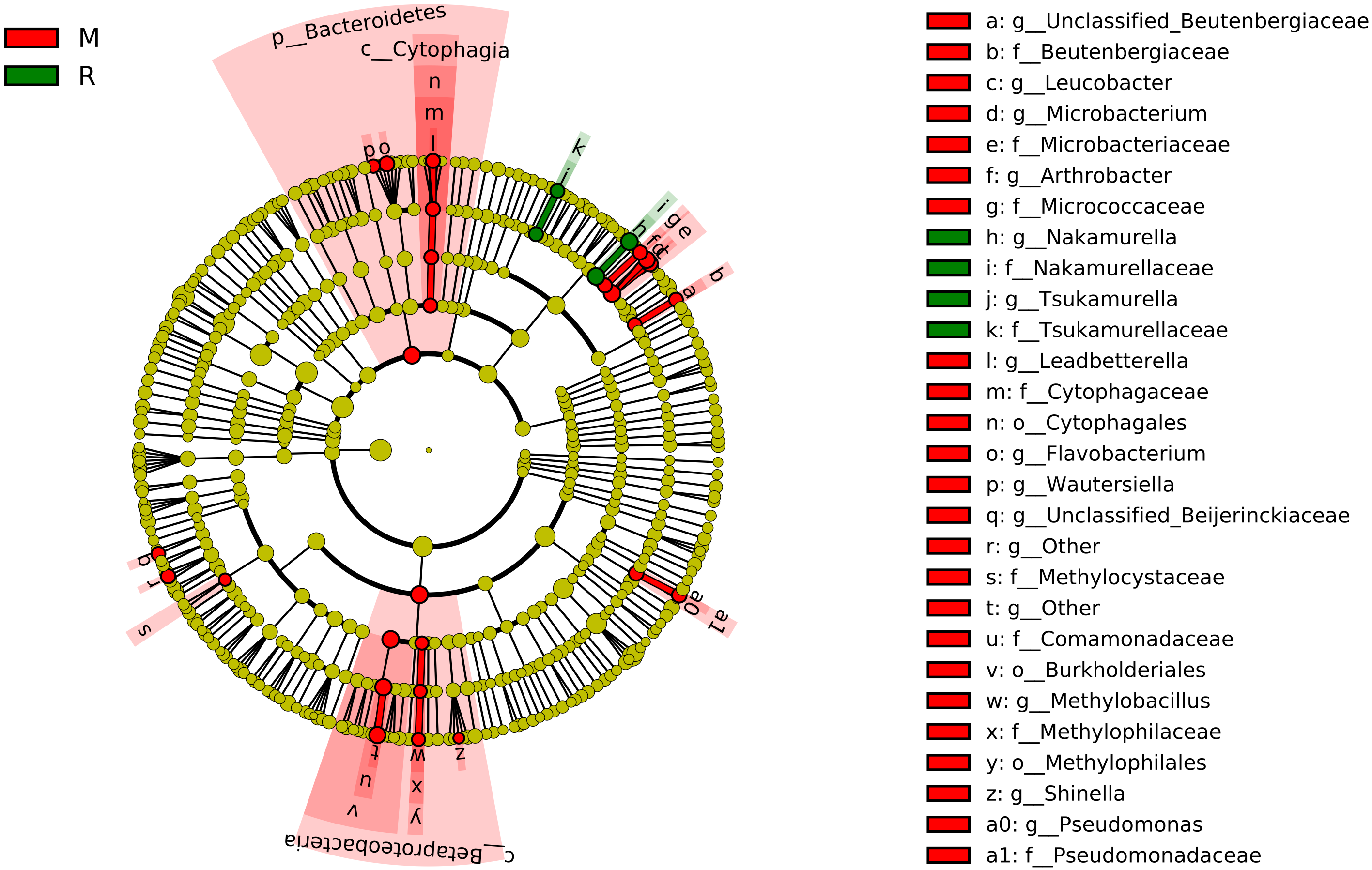

M
R
p__Bacteroidetes
c__Cytophagia
c__Betaproteobacteria
a: g__Unclassified_Beutenbergiaceae
b: f__Beutenbergiaceae
c: g__Leucobacter
d: g__Microbacterium
e: f__Microbacteriaceae
f: g__Arthrobacter
g: f__Micrococcaceae
h: g__Nakamurella
i: f__Nakamurellaceae
j: g__Tsukamurella
k: f__Tsukamurellaceae
l: g__Leadbetterella
m: f__Cytophagaceae
n: o__Cytophagales
o: g__Flavobacterium
p: g__Wautersiella
q: g__Unclassified_Beijerinckiaceae
r: g__Other
s: f__Methylocystaceae
t: g__Other
u: f__Comamonadaceae
v: o__Burkholderiales
w: g__Methylobacillus
x: f__Methylophilaceae
y: o__Methylophilales
z: g__Shinella
a0: g__Pseudomonas
a1: f__Pseudomonadaceae
