# Supplementary figures and images for: Effects of Host Plant and Insect Generation on Shaping of the Gut Microbiota in the Rice Leaffolder, Cnaphalocrocis medinalis
Source: Front Microbiol. 2022 Apr 11;13:824224. doi: 10.3389/fmicb.2022.824224 (PMC9037797; doi:10.3389/fmicb.2022.824224)

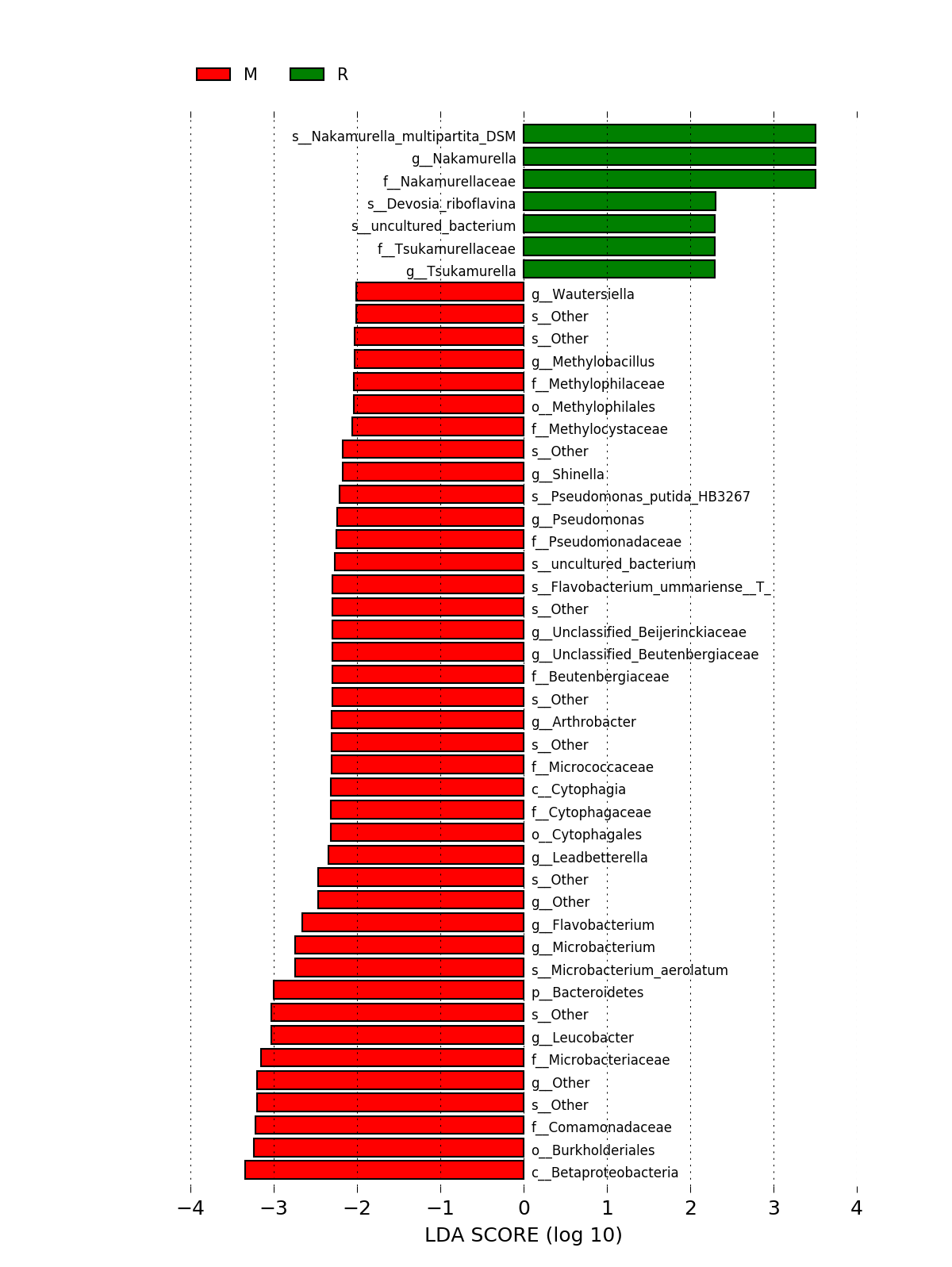

Supplement: Supplementary Figure 1 — Bacterial taxa with linear discriminant analysis (LDA) score >2 in the gut microbiota of C. medinalis fed on different host plants. [file Data_Sheet_1.ZIP › Supplementary Figure 1.tif]
